# The Fifteenth Annual Meeting

**Published:** 1855-07

**Authors:** Charles Bonsall


### THE FIFTEENTH ANNUAL MEETING

*Of the American Society of Dental Surgeons, convened according to Appointment, in the Hall of the Ohio Dental College, in the city of Cincinnati, Ohio, on Thuesday, May 8th,* 1855.

Met at 10 o'clock A. M., the President in the chair.

Present Drs. Townsend, Dunning, Wheeler, Miller, Berry, Goddard, Taylor, Allen and Bonsall, together with many other members of the profession ; several of them being members of the Mississippi Valley Association of Dental Surgeons, who had met for the purpose of welcoming this society.

In the absence of the Secretary, Dr. Bonsall was chosen Secretary *pro tem.*

On motion, all members of the profession who may be present at any of our sessions, were now invited to participate in our discussions.

The minutes of the last Annual Meeting were read by the Secretary, and approved, after which the President Dr. Townsend delivered the opening address which was listend to with much attention.

Dr. Townsend reported that the Chairman of the committee on Microscopic Observations, Dr. J. D. White had a report to make, but had been unavoidably prevented from attending the meeting; and on motion the committee was continued to report at the next Annual Meeting.

On motion of Dr. Taylor a committee of three was appointed to take into consideration, the suggestion made in the President's address; and Drs. Taylor, Dunning and Goddard were appointed and directed to report at the next session.

The Chair appointed Drs. Goddard and Taylor to audit the Treasurer's accounts.

Adjourned to meet at 9 o'clock A. M. to-morrow.

MORNING SESSION, SECOND DAY.

Met according to adjournment at 9 o'clock A. M., with the President in the Chair, the minutes of yesterday's meeting were read and approved.

The committee appointed to audit the Treasurer's accounts reported the following:

Your committee appointed to audit the accounts of the Treasurer, would respectfully report, that they have examined the same with the vouchers, and find them correct, and that there is a balance of $227,74 in his hands. All of which is respectfully submitted.

WM. H. GODDARD<br>

JAMES TAYLOR } Committee.

The next business in order being the report of the committee on the President's address, they offered the following report which was accepted.

*To the American Society, &c.*

Your committee to whom was referred the President's address, would respectfully report:

That they have especially turned their attention to that part of the address which suggests a more liberal—less exclusive, and more national organization: one which shall bring up at our yearly convocations a fair representation from every portion of our extended country.

The committee feel that the profession occupies now a very different position from that which it did when this society was first organized;

That however admirably adapted the organization was then for the work assigned it, yet having accomplished, as we believe, that work, and been of vast service to the profession—a broader basis for action is now demanded.

The Society was organized before we had a dental literature or any effective and well organized system of dental education. In the brief period of its existence, the work of a century has been accomplished, and the impetus, which has wrought such progress, demands a new set of machinery, that we may keep pace with the active spirit of the age.

The committee would therefore recommend that a call be issued by the President of this society, in accordance with the sixth article of the Constitution "To take into consideration the general subject of associations, and the dissolution of this society; said meeting to be held in Philadelphia, on the day previous to the holding of the meeting called by a number of dentists of that city."

JAMES TAYLOR<br>

E. J. DUNNING } Committee.<br>

WM. H. GODDARD

The subject was fully discussed by Drs. Miller, Dunning, Taft, Townsend, Taylor, Leslie, Pease, Watt and Goddard, and the resolutions were unanimously adopted.

On Motion of Dr. Goddard the President was requested to give *this afternoon*, to the members, who may be present, a description of his manner of plugging teeth.

Dr. Taylor reported the sudden death of Dr. B. N. Freeman, a member of the profession in attendance on the American Society, now in session in this city. Therefore,

*Resolved*, That we deplore the loss thus sustained by the profession, and deeply sympathize with his afflicted family in this sad and unexpected bereavement.

*Resolved*, That, as it was the will of Divine Providence that our friend and brother should die away from his wife and family, deprived of those family comforts so necessary to soothe the passage to the grave, we feel grateful that it was in our power to administer to his wants in the hour of his death. That he died not among strangers but was surrounded by friends.

*Resolved*, That the above be entered on our minutes and that the Secretary transmit a copy of the same to the family of the deceased.

James Taylor.

Wm. H. Goddard.

E. D. Wheeler.

On motion adjourned to meet at 3½ o'clock P. M.

AFTERNOON SESSION.

Met according to adjournment at 3½ o'clock P. M., when the minutes of the morning session were read and approved.

The President spent some time, apparently very much to the satisfaction of members, in detailing his method of plugging various kinds of cavities in teeth, and under many different circumstances, after which there was a general discussion on the subject of plugging, participated in by Drs. Miller, Bonsall, Taylor, Wheeler and others.

Adjourned till 9 o'clock A. M. to-morrow.

THIRD DAY, MORNING SESSION.

Met at 9 o'clock A. M., according to adjournment.

The minutes of last session were read and adopted.

On motion of Dr. Goddard the election of officers was made the special order of business for 11 o'clock this morning.

On motion, Dr. Townsend was requested to read to the society such portions of two lectures delivered by him to the dental class in the Philadelphia College as he may think proper.

This request was complied with, after which it was, on motion of Dr. Taylor,

*Resolved*, That the lectures read by our President this day on professional courtesy, &c. be requested for publication in the "Dental Register."

Drs. Goddard and Dunning being appointed as a nominating committee, reported that under the circumstances it was thought best to continue the present board of officers in office until the next meeting—said report was accepted and unanimously approved.

Dr. Dunning reported having found accidentally a much superior sharpening and polishing stone to the Arkansas stone; it is called Missouri Oil Stone.

The subject of separating the bicuspid teeth, with the best form of space to be left, was now spoken to by Drs. Dunning, Miller, Bishop, Webster and others.

Adjourned to meet at 3 o'clock P. M.

AFTERNOON SESSION.

Met at 3 o'clock P. M., when the subject of removing the first or second bicuspid or first morlar, for the purpose of giving room, where there was a prospect of a crowded dentition was now discussed by Drs. Dunning, Bonsall, Goddard, Taylor, Robbins, Smith, Wheeler, Miller, Bishop, Webster and others.

On motion the Treasurer was directed to pay the Janitor of the Ohio Dental College $8 for services.

Adjourned to meet at the residence of Dr. Bonsall, at 8 o'clock this evening.

EVENING SESSION.

Met according to adjournment at the residence of Dr. Bonsall, together with several members of the medical profession, and others, and held a general *Tooth talk.*

On motion of Dr. Dunning, it was

*Resolved*, That the thanks of the American Society of Dental Surgeons be, and they hereby are tendered to our brethren of the Mississippi Valley Association, and to the officers of the Ohio College of Dental Surgery for the courtesy and kindness extended to us during our sessions in Cincinnati.

On motion of Dr. Bonsall,

*Resolved*, That the thanks of this society be tendered to the President, Dr. Townsend, for the able, efficient and impartial manner in which he has filled the chair during our sessions.

On motion, Drs. Bonsall and Taylor were appointed a committee to publish the proceedings of the meeting.

Adjourned *sine die.*

Charles Bonsall,
Corresponding and Recording Secretary *pro tem.*

